# Preliminary evidence of imaging of chemokine receptor-4-targeted PET/CT with [^68^Ga]pentixafor in non-Hodgkin lymphoma: comparison to [^18^F]FDG

**DOI:** 10.1186/s13550-020-00681-7

**Published:** 2020-08-05

**Authors:** Qingqing Pan, Yaping Luo, Yan Zhang, Long Chang, Ji Li, Xinxin Cao, Jian Li, Fang Li

**Affiliations:** 1 Department of Nuclear Medicine, Chinese Academy of Medical Sciences and Peking Union Medical College Hospital, Beijing, People’s Republic of China; 2 Beijing Key Laboratory of Molecular Targeted Diagnosis and Therapy in Nuclear Medicine, Beijing, 100730 People’s Republic of China; 3 Department of Hematology, Chinese Academy of Medical Sciences and Peking Union Medical College Hospital, Beijing, People’s Republic of China; 4 Department of Gastroenterology, Chinese Academy of Medical Sciences and Peking Union Medical College Hospital, Beijing, People’s Republic of China

**Keywords:** Lymphoma, CXCR4, [^68^Ga]pentixafor, PET/CT

## Abstract

**Background:**

In order to study the CXCR4 expression with [^68^Ga]pentixafor PET in different types of non-Hodgkin lymphoma, we performed a retrospective study to describe the [^68^Ga]pentixafor PET/CT imaging in a spectrum of lymphomas and to compare it with [^18^F]FDG PET/CT.

**Results:**

Twenty-seven patients with newly diagnosed non-Hodgkin lymphoma were recruited retrospectively. [^68^Ga]pentixafor PET showed increased radioactivity in lymphoplasmacytic lymphoma (*n* = 8), marginal zone lymphoma (*n* = 4), diffuse large B cell lymphoma (*n* = 3), follicular lymphoma (*n* = 2), mantle cell lymphoma (*n* = 1), unclassified indolent B cell lymphoma (*n* = 3), and enteropathy associated T cell lymphoma (*n* = 3). However, peripheral T cell lymphoma, not otherwise specified (*n* = 1), and NK/T cell lymphoma (*n* = 2) were not avid for [^68^Ga]pentixafor. In comparison to [^18^F]FDG PET, [^68^Ga]pentixafor PET demonstrated more extensive disease and higher radioactivity in lymphoplasmacytic lymphoma and marginal zone lymphoma.

**Conclusion:**

CXCR4 expression varies in different types of non-Hodgkin lymphoma. Overexpression of CXCR4 was detected with [^68^Ga]pentixafor PET/CT in lymphoplasmacytic lymphoma, marginal zone lymphoma, diffuse large B cell lymphoma, follicular lymphoma, mantle cell lymphoma, unclassified indolent B cell lymphoma, and enteropathy associated T cell lymphoma. The uptake of [^68^Ga]pentixafor was higher than [^18^F]FDG in lymphoplasmacytic lymphoma and marginal zone lymphoma.

## Background

C-X-C motif chemokine receptor 4 (CXCR4) is a member of the G-protein coupled chemokine receptor family that mediates hemopoietic cell proliferation, migration, homing, and cell adhesion to extracellular matrix molecules. CXCR4 is physiologically expressed on T and B lymphocytes, monocytes, macrophages, neutrophils, eosinophils, and hematopoietic stem cells in bone marrow [1]. Pathological CXCR4 overexpression has been reported in more than 30 various types of solid tumors and hematopoietic malignancies. It plays a crucial role in tumor growth, progression, invasiveness, cancer cell-microenvironment interaction, and metastasis [2, 3].

[^68^Ga]pentixafor, a CXCR4-targeted PET probe with high affinity and selectivity to the receptor, has been developed and allows the sensitive and high-contrast PET imaging of CXCR4-expressing tissues and diseases in vivo [1]*.* The first clinical application of [^68^Ga]pentixafor PET has been carried out in patients with non-Hodgkin lymphoma and multiple myeloma, which confirmed the CXCR4 expression in these lymphoproliferative diseases as a proof-of-concept [4]. Since then, most studies of [^68^Ga]pentixafor were focused on evaluation of hematologic malignancies, for example, multiple myeloma [1, 5–7], Waldenström macroglobulinemia/lymphoplasmacytic lymphoma [8, 9], mucosa associated lymphoid tissue (MALT) lymphoma [10], chronic lymphocytic leukemia [11], and acute myeloid leukemia [12, 13]. Some studies showed remarkable superiority of [^68^Ga]pentixafor PET in detecting tumors and staging of the disease when compared with [^18^F]FDG PET [5, 7, 8]. Besides the diagnostic use of [^68^Ga]pentixafor PET, CXCR4-directed radioligand therapy with ^90^Y- or ^177^Lu-labeled Pentixather, the therapeutic partner of [^68^Ga]pentixafor, has been successfully introduced as the compassionate use of treatment for relapsed, advanced stage multiple myeloma, diffuse large B cell lymphoma (DLBCL), and acute myeloid leukemia, in addition to high-dose chemotherapy regimens and followed by subsequent hematopoietic stem cell transplantation [13–16].

These studies depict the potential of CXCR4-directed PET imaging and radioligand therapy in hematologic malignancies. Considering the biologic differences of each tumor entity that may result in various levels of CXCR4 expression, it is necessary to study the CXCR4 expression with [^68^Ga]pentixafor PET to help better select the patient for CXCR4-directed imaging and personalized therapy in future clinical applications. Herein, we performed this retrospective study to describe the [^68^Ga]pentixafor PET/CT imaging in a spectrum of non-Hodgkin lymphomas and to compare it with [^18^F]FDG PET/CT, which served as a reference.

## Methods

### Patients

Between 2016 and 2019, twenty-seven patients with newly diagnosed non-Hodgkin lymphoma that underwent both [^68^Ga]pentixafor and [^18^F]FDG PET/CT in a clinical trial (NCT 03436342) were recruited. [^18^F]FDG and [^68^Ga]pentixafor PET/CT were carried out within 1 week. The imaging characteristics were analyzed, and quantitative parameters were measured retrospectively. The study was approved by the institutional review board of PUMCH (IRB protocol #ZS-1113), and written informed consent was obtained from each patient.

### PET/CT imaging

The DOTA-CPCR4-2 peptide was purchased from CSBio Co (CA 94025, USA). The radiolabeling of [^68^Ga]pentixafor was performed manually before injection according to the procedures as previously published [8]. [^18^F]FDG was synthesized in house with an 11 MeV cyclotron (CTI RDS 111, Siemens, Germany).

The PET scans were performed on dedicated PET/CT scanners (Biograph64 Truepoint TrueV, Siemens, Germany; Polestar m660, SinoUnion, China) from the tip of the skull to the middle thigh. For [^18^F]FDG PET/CT, patients fasted for over 6 h and the blood glucose levels were monitored (4.4–8.8 mmol/L) prior to an injection of [^18^F]FDG (5.55 MBq/kg). The PET/CT images (2 min/bed) were acquired with an uptake time of 76.0 ± 15.8 min (range 50–105 min). For [^68^Ga]pentixafor PET/CT, imaging was performed (2–4 min/bed) with an uptake time of 56.1 ± 22.0 min (range 30–108 min) after injection of 2.8 ± 0.9 MBq (range 1.3–5.0 MBq) [^68^Ga]pentixafor. All patients underwent unenhanced low-dose CT (120 kV, 30–50 mAs) for attenuation correction and anatomical reference. The acquired data were reconstructed using the ordered subset expectation maximization method (Siemens Biograph 64 2 iterations, 8 subsets, Gaussian filter, image size 168 × 168; SinoUnion Polestar 2 iterations, 10 subsets, Gaussian filter, image size 192 × 192).

### Image analysis and statistics

All PET/CT scans were first visually rated in a binary fashion by 2 experienced nuclear medicine physicians (YL and QP), both of whom had over 7 years and 2 years experience in reading [^18^F]FDG and [^68^Ga]pentixafor PET/CT, respectively. Lesions were visually determined as focally increased tracer retention as compared to surrounding normal tissue. As previously described [7, 8], bone marrow involvement in PET/CT was interpreted as being positive if there was presence of focal lesions with positive PET results, or diffuse bone marrow patterns with uptake higher than liver. The involvement of lymphoma and intensity of lesions were recorded. Visually, the tracer depicting higher number or intensity of tumor lesions was considered superior. Semi-quantitative analysis was performed with standard uptake value (SUV) and tumor-to-background ratios (TBR). A circular region of interest was placed over the tumor area with the maximal radioactivity, and SUVmax of the lesion was generated. Tumor uptakes were presented as SUVmax of the most avid lesion in a patient or in an organ. A reference blood pool region was defined by drawing a region of interest in the left atrium of the heart to derive TBR_blood_, and region of interest was drawn in liver to derive TBR_liver_.

Statistical analyses were done with the SPSS Statistics software (version 22.0, IBM SPSS Inc.). Comparison of numerical data of 2 groups was performed using Student’s *t* test for data with normal distribution and Wilcoxon rank sum test for skewed data. A *p* value < 0.05 was considered statistically significant.

## Results

**Fig. 1:**
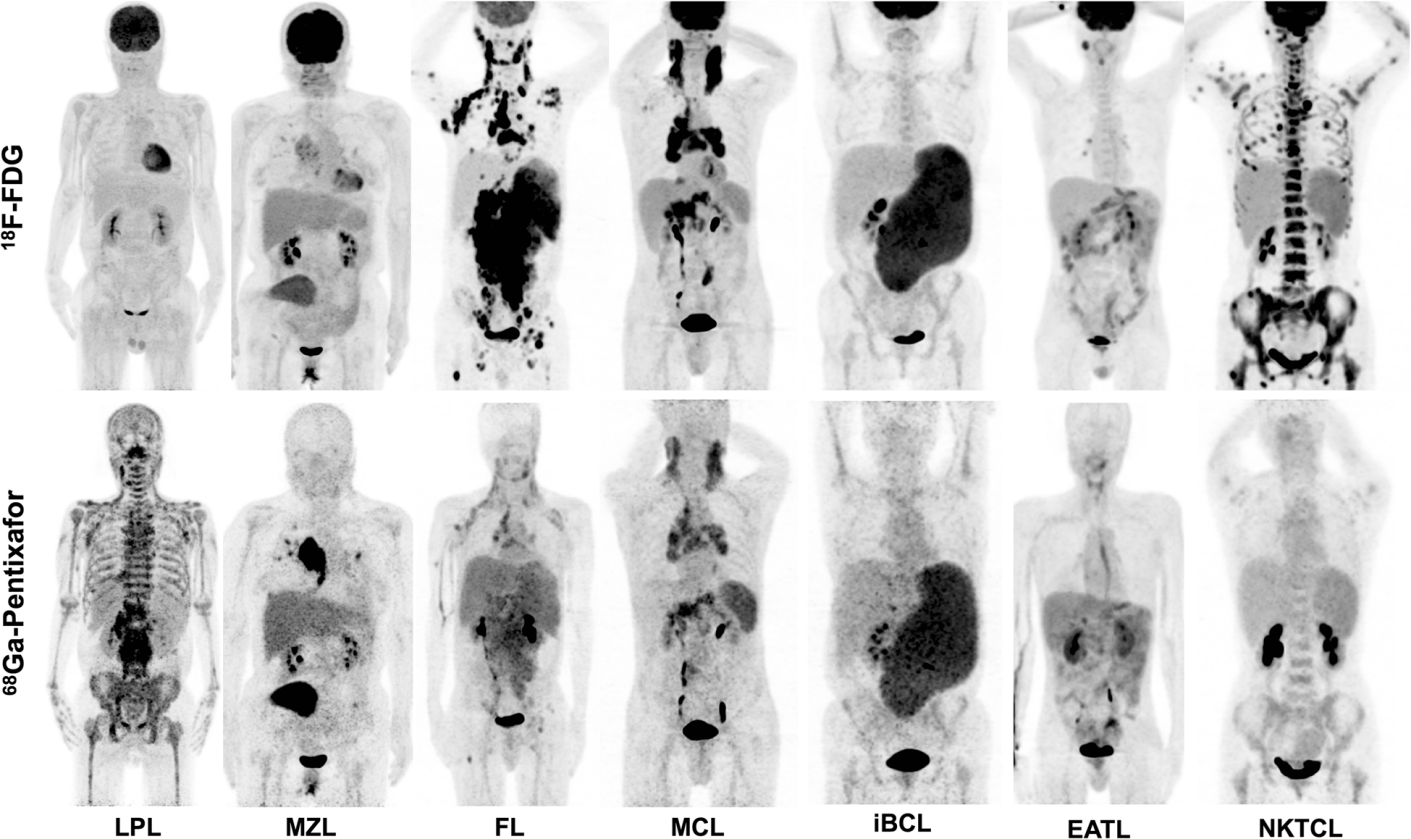
Individual comparison of lymphomas shown on ^18^F-FDG and ^68^Ga-Pentixafor PET/CT. ^68^Ga-Pentixafor PET showed obviously higher intensity than ^18^F-FDG uptake in lymphoplasmacytic lymphoma (LPL) and marginal zone lymphoma (MZL). ^68^Ga-Pentixafor PET also detected more disease involvement in bone marrow, lymph nodes, and paramedullary disease in LPL. ^68^Ga-Pentixafor PET showed increased accumulation of radioactivity in follicular lymphoma (FL) and mantle cell lymphoma (MCL), but was lower than the ^18^F-FDG uptake. ^68^Ga-Pentixafor and ^18^F-FDG PET detected disease involvement with comparable uptake of both tracers in unclassified indolent B cell lymphoma (iBCL) and enteropathy associated T cell lymphoma (EATL). NK/T cell lymphoma (NKTCL) was very ^18^F-FDG-avid but was negative of ^68^Ga-Pentixafor uptake

**Fig. 2:**
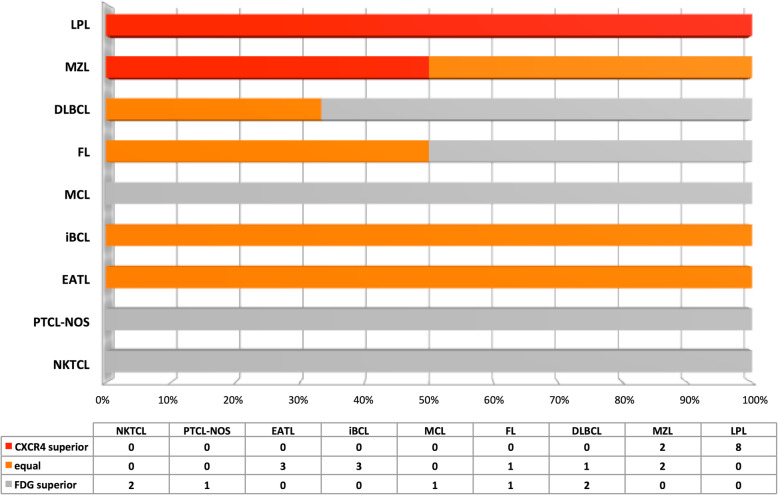
Visual comparison of lymphomas shown on ^68^Ga-Pentixafor and ^18^F-FDG PET/CT. LPL, lymphoplasmacytic lymphoma; MZL, marginal zone lymphoma; DLBCL, diffuse large B cell lymphoma; FL, follicular lymphoma; MCL, mantle cell lymphoma; iBCL, indolent B cell lymphoma; EATL, enteropathy associated T cell lymphoma; PTCL-NOS, peripheral T cell lymphoma, not otherwise specified; NKTCL, NK/T cell lymphoma

The lymphomas of the 27 enrolled patients (19 male, 8 female; age, 57.2 ± 13.1 years, range 15–76 years) included lymphoplasmacytic lymphoma (*n* = 8), marginal zone lymphoma (*n* = 4), peripheral T cell lymphoma (*n* = 4), DLBCL (*n* = 3), unclassified indolent B cell lymphoma (*n* = 3), follicular lymphoma (*n* = 2), NK/T cell lymphoma (*n* = 2), and mantle cell lymphoma (*n* = 1). The patients did not receive any treatment against lymphoma before PET/CT scans. Clinical characteristics of the recruited patients and the diagnostic performance of dual-tracer PET/CT in each case are given in Table 1, and examples of maximum intensity projections of the dual-tracer PET scans in lymphomas are shown in Fig. 1. The semiquantitative and visual comparisons of the lymphoma detected in [^68^Ga]pentixafor and [^18^F]FDG PET/CT are shown in Table 2 and Fig. 2, respectively.

**Table 1:** Patients’ clinical characteristics and PET/CT diagnosis

No.	Age/sex	Type of lymphoma	Involvement	PET/CT diagnosis	
				CXCR4	^18^F-FDG
1	59/F	DLBCL	Cerebrum, ethmoidal sinus	P	P
2	32/M	FL	LN, BM, spleen, lung	P	P
3	53/M	iBCL	LN, BM, spleen	P	P
4	60/M	DLBCL	Ileum	P	P
5	64/M	EATL	Intestines	P	P
6	50/M	EATL	Small intestines, BM	N	N
7	60/F	iBCL	BM, spleen	P	P
8	70/M	iBCL	BM, spleen	P	P
9	71/M	MCL	LN	P	P
10	64/M	DLBCL	Thyroid	P	P
11	41/F	NKTCL	Nasal cavity, pharynx, LN, subcutaneous, BM	N	P
12	52/F	NKTCL	Paranasal sinus, orbit, cerebrum	N	P
13	15/M	PTCL-NOS	Musculature	N	P
14	51/M	MZL	Lung	P	P
15	65/F	MZL	Cerebral dura mater, kidney, retroperitoneum	P	P
16	71/F	MZL	Lung, subcutaneous	P	P
17	67/F	LPL	BM	P	N
18	59/M	FL	LN	P	P
19	58/M	LPL	BM, LN, liver, pancreas, PMD	P	P
20	48/M	LPL	BM, LN, liver, PMD	P	P
21	62/M	LPL	BM, LN	P	P
22	74/M	EATL	Small intestines	P	P
23	76/M	LPL	BM, LN	P	N
24	56/F	MZL	Spleen	P	P
25	51/M	LPL	BM, LN, PMD, pleura	P	N
26	53/M	LPL	BM, LN	P	P
27	64/M	LPL	BM, LN	P	P

*DLBCL* diffuse large B cell lymphoma, *FL* follicular lymphoma, *iBCL* indolent B cell lymphoma, *EATL* enteropathy associated T cell lymphoma, *MCL* mantle cell lymphoma, *NKTCL* NK/T cell lymphoma, *PTCL-NOS* peripheral T cell lymphoma, not otherwise specified, *MZL* marginal zone lymphoma, *LPL* lymphoplasmacytic lymphoma, *LN* lymph nodes, *BM* bone marrow, *PMD* paramedullary disease

**Table 2:** Comparison of SUVmax and TBR in lymphomas

Type of lymphoma	SUVmax		TBR_blood_		TBR_liver_	
	CXCR4	^18^F-FDG	CXCR4	^18^F-FDG	CXCR4	^18^F-FDG
LPL (*n* = 8)	11.6 ± 3.2	3.2 ± 0.8	6.5 ± 2.8	2.2 ± 0.7	4.5 ± 1.1	1.2 ± 0.3
MZL (*n* = 4)	12.1 ± 5.0	5.3 ± 1.2	6.2 ± 4.1	2.5 ± 0.6	4.2 ± 1.1	1.5 ± 0.4
DLBCL (*n* = 3)	4.8 ± 1.7	14.9 ± 3.6	2.8 ± 0.9	9.5 ± 3.6	2.0 ± 0.8	6.1 ± 3.1
FL (*n* = 2)	7.6 ± 3.5	16.7 ± 0.5	5.4 ± 4.2	14.2 ± 6.6	4.1 ± 3.1	8.5 ± 3.1
MCL (*n* = 1)	6.2	10.1	3.0	5.3	3.6	4.0
iBCL (*n* = 3)	4.3 ± 1.8	4.2 ± 1.5	1.9 ± 1.6	2.3 ± 1.9	1.6 ± 0.3	1.7 ± 0.8
PCTL-NOS (*n* = 1)	1.3	7.8	1.0	4.9	1.2	3.4
ETAL (*n* = 3)	3.1 ± 1.9	2.2 ± 2.0	1.7 ± 1.1	1.6 ± 1.0	1.7 ± 0.9	1.1 ± 0.9
NKTCL (*n* = 2)	3.6 ± 0.1	14.2 ± 7.5	1.3 ± 0.0	10.1 ± 6.9	1.4 ± 0.2	5.5 ± 2.9

### Lymphoplasmacytic lymphoma

Bone marrow is the predominant site of involvement in lymphoplasmacytic lymphoma, which was confirmed by bone marrow aspiration and biopsy in all recruited patients. On [^68^Ga]pentixafor PET/CT, all 8 patients had intense radioactivity in the bone marrow, with an SUVmax of 9.5 ± 2.6 (range, 7.0–14.8). With [^18^F]FDG PET/CT, the bone marrow intensity was comparable to the uptake in liver (SUVmax 2.1–4.6). In comparisons of [^68^Ga]pentixafor and [^18^F]FDG, all 8 patients had visually higher uptake in the bone marrow on [^68^Ga]pentixafor PET than on [^18^F]FDG PET. Regarding the extent of bone marrow involvement, [^68^Ga]pentixafor PET demonstrated more extensive bone marrow disease in 6 patients than [^18^F]FDG PET, specifically when the involvement of the craniofacial bones and distal upper extremity bones was visualized.

[^68^Ga]pentixafor PET/CT detected positive lymph node involvements in 7 patients (SUVmax 11.3 ± 3.5, range 6.9–16.9), and 6 patients had involvement in more than 5 lymph node regions. However, with [^18^F]FDG PET/CT, only 3 patients were found to have mildly FDG-avid lymph nodes (SUVmax 1.2–4.7). Moreover, [^68^Ga]pentixafor PET/CT detected more positive lymph nodes with higher radioactivity in these 3 patients than [^18^F]FDG PET/CT. Additionally, [^68^Ga]pentixafor detected disease of paramedullary involvements, liver, pancreas, and pleura in 3 patients; however, the above lesions were missed in [^18^F]FDG PET. The SUVmax and TBR of the matched disease in bone marrow, lymph node, and other involvements were significantly higher in [^68^Ga]pentixafor PET than in [^18^F]FDG PET (paired Student’s *t* test, *p* < 0.01).

### Marginal zone lymphoma

Four patients were confirmed with marginal zone lymphoma at histology, including 3 patients with MALT lymphoma and 1 patient with splenic marginal zone lymphoma. The 3 patients with MALT lymphoma had disease involved the lung, kidney, retroperitoneum, subcutaneous area, and cerebral dura mater. [^68^Ga]pentixafor PET/CT showed intense radioactivity in the above lesions, with an SUVmax of 13.2 ± 4.1 (range, 8.9–20.3). With [^18^F]FDG PET/CT, the MALT lymphoma involvements were hypermetabolic (SUVmax 4.1–5.5), but the uptake and TBR of [^18^F]FDG were significantly lower than that of [^68^Ga]pentixafor (paired Student’s *t* test, *p* < 0.05). Furthermore, [^68^Ga]pentixafor PET also detected disease in cerebral dura mater with intense [^68^Ga]pentixafor uptake in one patient, which was not shown in [^18^F]FDG PET. The patient with splenic marginal zone lymphoma had solitary disease in the spleen, which showed comparative uptake of both tracers (SUVmax, [^68^Ga]pentixafor vs. [^18^F]FDG, 5.5 vs. 7.5).

### Diffuse large B cell lymphoma

Three patients were diagnosed with DLBCL affecting the cerebrum, ethmoid sinus, ileum, and thyroid. [^68^Ga]pentixafor PET/CT showed increased uptake of [^68^Ga]pentixafor in these lesions; however, the intensity of radioactivity in [^68^Ga]pentixafor PET was significantly lower than that in [^18^F]FDG PET (SUVmax, [^68^Ga]pentixafor vs. [^18^F]FDG, 4.8 ± 1.7 vs. 14.9 ± 3.6, *p* = 0.030). It is noteworthy that although the uptake of [^68^Ga]pentixafor in the cerebral DLBCL was lower than the uptake of [^18^F]FDG in this lesion, the visual assessment of the disease in [^68^Ga]pentixafor PET and [^18^F]FDG PET was comparable due to the low background radioactivity of [^68^Ga]pentixafor in the brain (TBR in [^68^Ga]pentixafor PET is much higher than the TBR in [^18^F]FDG PET if normal cerebrum regarded as background [TBR, 10.8 vs. 1.6]).

### Follicular lymphoma

The 2 patients with follicular lymphoma had disease involved the lymph nodes, spleen, lung, and bone marrow. In one patient with follicular lymphoma (WHO grades 1–2) involving a few lymph nodes, the radioactivity of the lesions and the extension of the disease detected in [^68^Ga]pentixafor PET and [^18^F]FDG PET were similar. In another patient with follicular lymphoma (WHO grade 3B) that was extensively involved the lymph nodes, spleen, lung, and bone marrow, the [^68^Ga]pentixafor uptake in the lymphoma lesions was increased; however, it was much lower than the [^18^F]FDG uptake in these lesions (SUVmax, [^68^Ga]pentixafor vs. [^18^F]FDG, 5.1 vs. 17.0).

### Mantle cell lymphoma

Only one patient had mantle cell lymphoma, which involved multiple lymph node regions. The involved lymph nodes had moderately increased uptake of [^68^Ga]pentixafor (SUVmax 6.2), but was lower than the uptake intensity of [^18^F]FDG (SUVmax 10.1).

### Indolent B cell lymphoma, unclassified

Unclassified indolent B cell lymphoma was confirmed in 3 patients, who had involvement in bone marrow, spleen, and a few lymph nodes. Both [^68^Ga]pentixafor PET and [^18^F]FDG PET showed mild to moderate radioactivity in the lymphoma involvements.

### Peripheral T cell lymphoma

Three patients were diagnosed with enteropathy associated T cell lymphoma (EATL). In 2 patients with EATL, both [^68^Ga]pentixafor PET and [^18^F]FDG PET showed mildly increased radioactivity in the intestines; however, the dual-tracer PET/CT results were both false negative in the remaining one patient with EATL. One patient had peripheral T cell lymphoma, not otherwise specified (PTCL-NOS) involving musculature. The disease had intense uptake of [^18^F]FDG (SUVmax 7.8); however, it was not avid for [^68^Ga]pentixafor (SUVmax 1.3).

### NK/T cell lymphoma

Two patients were diagnosed with NK/T cell lymphoma, affecting the nasal cavity, paranasal sinus, orbit, cerebrum, pharynx, lymph node, subcunaneous area, and bone marrow. The uptake of [^68^Ga]pentixafor in these lesions was not increased; meanwhile, the lesions were very FDG-avid (SUVmax, [^68^Ga]pentixafor vs. [^18^F]FDG, 3.6 vs. 19.5).

## Discussion

Our study demonstrated overexpression of CXCR4 in several types of non-Hodgkin lymphoma with [^68^Ga]pentixafor PET/CT, including lymphoplasmacytic lymphoma, marginal zone lymphoma, DLBCL, follicular lymphoma, mantle cell lymphoma, unclassified indolent B cell lymphoma, and EATL. Notably, [^68^Ga]pentixafor PET might be superior to [^18^F]FDG PET in detecting the disease with higher radioactivity in lymphoplasmacytic lymphoma and marginal zone lymphoma. However, the recruited patients with PTCL-NOS and NK/T cell lymphoma were negative on [^68^Ga]pentixafor PET, although the disease was very FDG avid.

Lymphoplasmacytic lymphoma is an indolent non-Hodgkin lymphoma characterized by the accumulation of lymphoplasmacytic cells producing excessive monoclonal immunoglobulin in the bone marrow. As lymphoplasmacytic lymphoma is usually not avid for [^18^F]FDG [17], [^18^F]FDG PET/CT is not indicated for lymphoplasmacytic lymphoma unless there is a suspicion of aggressive transformation [18]. Consistent with our previous research [8, 9], the current study again revealed an obvious superiority of [^68^Ga]pentixafor to [^18^F]FDG in staging of lymphoplasmacytic lymphoma. These results observed in clinical investigation were also supported by the fact that the CXCR4 expression is higher in the B cells of patients with lymphoplasmacytic lymphoma than in the B cells of healthy donors [19]. Therefore, the CXCR4-targeted PET/CT imaging with [^68^Ga]pentixafor may become an important tool for diagnosis and staging of lymphoplasmacytic lymphoma.

The staging of marginal zone lymphoma is a challenge with [^18^F]FDG PET/CT, because marginal zone lymphoma usually does not present with elevated glycolysis and may had heterogeneous metabolic behavior [20, 21]. Stollberg S. and the colleagues found that CXCR4 expression was present at a high intensity in 92% of the cases [22]. In a recent study of MALT lymphoma, 33/36 patients were positive on [^68^Ga]pentixafor PET/MRI showing a high uptake of [^68^Ga]pentixafor [10]. Another head-to-head comparison study of [^68^Ga]pentixafor and [^18^F]FDG revealed that 95.2% of marginal zone lymphoma patients had positive lesions on [^68^Ga]pentixafor PET/CT, as compared to only 42.9% of the patients who were positive on [^18^F]FDG PET/CT [23]. In accordance with the above research, our study showed that the 4 patients with marginal zone lymphoma were detected by [^68^Ga]pentixafor PET/CT with markedly increased radioactivity, which was superior or at least equal to [^18^F]FDG PET/CT. This result indicates that [^68^Ga]pentixafor PET/CT might be useful in detection and staging of marginal zone lymphoma; however, further study in larger patient cohort is warranted.

A previous study determined that CXCR4 was highly expressed in DLBCL cell lines, and in a patient with DLBCL, [^68^Ga]pentixafor PET/CT resulted in excellent tumor uptake [4]. Due to the highly expressed CXCR4 in DLBCL, experimental CXCR4-directed radioligand therapy was also used as part of the conditioning regimen prior to allogeneic stem cell transplantation in several patients with relapsed advanced-stage DLBCL [16]. Our study added evidence to the overexpression of CXCR4 in DLBCL, although the uptake of [^68^Ga]pentixafor was lower than the FDG uptake. For the lesions within the brain, [^68^Ga]pentixafor exhibited a clear delineation with higher image contrast compared to [^18^F]FDG due to the negligible uptake in cerebrum.

Strong CXCR4 expression is also detected in follicular lymphoma with both flow cytometry and immunohistochemical analysis [24, 25]. We previously reported the findings of [^68^Ga]pentixafor PET/CT in a patient with post-treated POEMS syndrome and concurrent follicular lymphoma, with active uptake of [^68^Ga]pentixafor (SUVmax 9.7) [26]. The results of the current study corresponded well to the previous studies, and it suggested that [^68^Ga]pentixafor might be useful in evaluation of follicular lymphoma in future.

Mantle cell lymphoma is usually an FDG-avid lymphoma, and [^18^F]FDG PET/CT is a useful tool in staging and evaluating treatment response [27, 28]. For CXCR4 expression, studies found that mantle cell lymphomas displayed high levels of CXCR4 expression, which is critical for malignant B cell trafficking and homing to supportive tissue microenvironment [25, 29, 30]. Consistent with these results, the only one patient with mantle cell lymphoma in our study had increased uptake of [^68^Ga]pentixafor in the involved lymph nodes, despite the accumulation of [^68^Ga]pentixafor that was obviously lower than that of [^18^F]FDG. As there is no other clinical data on the presentation of [^68^Ga]pentixafor PET in mantle cell lymphoma, it is hard to draw to a conclusion based on this single case. We think it might be interesting to further investigate the role of [^68^Ga]pentixafor PET and the feasibility of CXCR4-directed radioligand therapy in mantle cell lymphoma.

There is no reported clinical data on the CXCR4-targeted imaging in T cell and NK/T cell lymphomas, except a single case report of mycosis fungoides showing intense uptake of [^68^Ga]pentixafor [31]. Weng AP and the colleagues reported that only 11.5% (3/26) of the cases with peripheral T cell lymphoma exhibited positive immunohistochemical staining for CXCR4 [32]. This low positive rate of CXCR4 expression explains the negative result of CXCR4-targeted PET imaging in the patient with PTCL-NOS in our study. Similarly, the two patients with NK/T cell lymphoma did not show increased uptake of [^68^Ga]pentixafor in the tumors, which were very FDG-avid, suggesting the lack of CXCR4 expression in NK/T cell lymphoma as well.

EATL is a rare and aggressive subtype of extranodal T cell lymphoma arising in the gastrointestinal tract. The status of CXCR4 expression in EATL has not been reported yet. In our study, 2 of the 3 patients with EATL were positive on [^68^Ga]pentixafor PET, although the uptake of [^68^Ga]pentixafor was mild (SUVmax 5.1). Unlike [^18^F]FDG, there is no physiological uptake of [^68^Ga]pentixafor in the gastrointestinal tract. Therefore, we think [^68^Ga]pentixafor might play some role in detecting lymphoma in the intestines.

Our study has several limitations. First, the study has a small sample size. In some types of lymphoma, the number of cases was too small to perform a head-to-head comparison of the detection rate in lymphoma between [^68^Ga]pentixafor and [^18^F]FDG. Second, we only include several types of lymphoma in our study. In addition to the lymphomas included in the current study, chronic lymphocytic leukemia, acute lymphoblastic leukemia, and acute myeloid leukemia have been reported being positive on [^68^Ga]pentixafor PET [4, 11–13]. In vitro studies with flow cytometry, reverse transcription-polymerase chain reaction, and immunohistochemical analysis also detected strong expression of CXCR4 in hairy cell leukemia, T cell lymphoblastic lymphoma/leukemia, Sézary syndrome, angioimmunoblastic lymphoma, and anaplastic large cell lymphoma [32–39]. The CXCR4-targeted imaging with [^68^Ga]pentixafor in these lymphoma/leukemia needs to be further investigated. Third, we did not perform the in vitro studies to confirm the expression of CXCR4 in the recruited cases. Previous studies have determined that [^68^Ga]pentixafor binded with high specificity and selectivity to human CXCR4 and CXCR4 expression was correlated with cellular uptake of [^68^Ga]pentixafor in lymphoma cell lines [4]. We think these results have provided strong evidence of the mechanism of CXCR4-mediated [^68^Ga]pentixafor uptake in lymphoma. Finally, the heterogeneity of PET/CT protocols (e.g., uptake time, administered activity, use of 2 different PET/CT scanners, and reconstruction parameters) may bias the quantitative PET/CT measurements.

## Conclusion

The CXCR4 expression varies in different types of non-Hodgkin lymphoma. Overexpression of CXCR4 was detected with [^68^Ga]pentixafor PET/CT in lymphoplasmacytic lymphoma, marginal zone lymphoma, DLBCL, follicular lymphoma, mantle cell lymphoma, unclassified indolent B cell lymphoma, and EATL. However, PTCL-NOS and NK/T cell lymphoma may not present CXCR4 overexpression. When comparing with [^18^F]FDG, [^68^Ga]pentixafor showed higher uptake than [^18^F]FDG did in lymphoplasmacytic lymphoma and marginal zone lymphoma.

## Data Availability

The datasets generated during and/or analyzed during the current study are available from the corresponding author on reasonable request.

## Abbreviations

CXCR4: C-X-C motif chemokine receptor 4
MALT: Mucosa associated lymphoid tissue
DLBCL: Diffuse large B cell lymphoma
SUV: Standard uptake value
TBR: Tumor-to-background ratios
EATL: Enteropathy associated T cell lymphoma
PTCL-NOS: Peripheral T cell lymphoma, not otherwise specified
